# Systemic inflammation modulates the ability of serum ferritin to predict all-cause and cardiovascular mortality in peritoneal dialysis patients

**DOI:** 10.1186/s12882-020-01892-9

**Published:** 2020-06-23

**Authors:** Sha Fu, Junzhe Chen, Bo Liu, Peifen Liang, Yuchun Zeng, Min Feng, Zhenjian Xu, Guiqiong Zheng, Suqiong Yang, Anping Xu, Ying Tang

**Affiliations:** Department of Nephrology, SunYat-Sen Memorial Hospital, Sun Yat-Sen University, 107 Yanjiang West Road, Guangzhou, 510080 China

**Keywords:** Inflammation, Serum ferritin, High-sensitivity C-reactive protein, Peritoneal dialysis, Mortality

## Abstract

**Background:**

This study aimed to ascertain whether the correlation of high serum ferritin with mortality is affected by systemic inflammation and to investigate the optimal serum ferritin level for predicting death when inflammation is considered in peritoneal dialysis (PD) patients.

**Methods:**

We classified 221 patients into four groups according to serum ferritin concentration (100 μg/L) and high-sensitivity CRP (hs-CRP) level (3 mg/L), and followed them regularly from the date of catheterization to Dec 31, 2016, at Sun Yat-Sen Memorial Hospital, China. Clinical and biochemical data were collected at baseline, and clinical outcomes such as all-cause and cardiovascular mortality were assessed.

**Results:**

During a median follow-up of 35 months (3 ~ 109 months), 50 (22.6%) deaths occurred. Cardiovascular disease (46.0%) was the most common cause of death, followed by infection (10.0%). The Kaplan–Meier survival analysis and log-rank test revealed significantly worse survival accumulation among PD patients with higher serum ferritin (≥100 μg/L) under elevated hsCRP levels (> 3 mg/L) (*P* = 0.022). A multivariate Cox regression analysis revealed that an increased serum ferritin level was independently associated with a higher risk of all-cause and cardiovascular mortality in PD patients (HR = 3.114, *P* = 0.021; and HR = 9.382, *P* = 0.032) with hsCRP above 3 mg/L after adjusting for relevant confounding factors.

**Conclusion:**

Higher serum ferritin levels were associated with an increased risk of all-cause and cardiovascular mortality in patients undergoing PD only in the presence of elevated hsCRP levels. The correlation of serum ferritin with poor outcome should take into consideration systemic inflammation.

## Background

Accumulating evidence has demonstrated that inflammation is negatively associated with the mortality and residual renal function of peritoneal dialysis (PD) patients. Serum ferritin is widely recognized as an acute phase reactant, that is nonspecifically enhanced under systemic inflammatory conditions, including chronic kidney disease (CKD), liver disease, and cancer [1–4]. Higher serum ferritin levels can induce macrophage accumulation and increase reactive oxygen species (ROS) formation during inflammation [5]. It was recently noted that serum ferritin concentration is highly correlated with mortality and cardiovascular outcome in maintenance hemodialysis patients (MHD) [6, 7]. However, the effect of serum ferritin on the long-term outcome of PD patients remains unclear.

Systemic inflammation is usually assessed by C-reactive protein (CRP) levels which have been widely considered to be associated with all-cause and cardiovascular mortality in patients undergoing dialysis [8]. High-sensitivity CRP (hs-CRP) is more sensitive than conventional CRP and a better marker for predicting 5-year mortality and dropout among PD patients [9, 10]. Recent studies have shown that persistent inflammation exacerbates the effects of risk factors that predict the poor outcome of CKD patients, such as serum albumin and osteoprotegerin [11, 12]. Therefore, we hypothesized that systemic inflammation might serve as a catalyst and modulate the effect of serum ferritin on the long-term prognosis of PD patients. We hence undertook this study to (1) ascertain the serum ferritin level that is negatively associated with the long-term survival of PD patients and (2) investigate whether the predictive role of serum ferritin is affected by hsCRP levels.

## Methods

### Patients

This retrospective, observational, cohort study enrolled 221 patients with end-stage kidney disease (ESKD) who underwent percutaneous PD catheter insertion in our hospital and had been undergoing PD more than 3 months. The PD modality was Continuous Ambulatory Peritoneal Dialysis (CAPD) using glucose-containing dialysis fluid which was exchanged four or five times daily. All patients with anemia were on erythropoiesis-stimulating agent (ESA) therapy and received iron supplementation (intravenous or oral iron) if transferrin saturation (TSAT) ≤30% and ferritin ≤500 μg/L. This study was conducted in the PD center of Sun Yat-sen Memorial Hospital, China, from October 2007 to December 2016. Patients with a duration of maintenance PD of less than 3 months were excluded.

The study and protocol were reviewed and approved by the Medical Ethics Committee of SunYat-Sen Memorial Hospital, Sun Yat-Sen University. We reviewed and followed up the medical data according to the guidelines of our ethics committee. Oral informed consents from PD patients were obtained if we followed up by telephone. The follow-up period was from the time of percutaneous PD catheter insertion to 31 December 2016. Patients were followed up to monitor deaths including all-cause and cardiovascular deaths. Cardiovascular mortality was defined as death from acute myocardial infarction, arrhythmia, heart failure, cerebral hemorrhage, cerebral infarction or sudden death.

### Data collection

Baseline demographic and clinical parameters, including age, gender, body mass index (BMI), primary renal disease, laboratory data, and dialysis information prior to the first PD session, were obtained from the medical records and recorded in a special PD registration form. Blood samples were drawn prior to PD catheter insertion at the time of hospitalization and were collected under the same conditions. Available laboratory data included 24 h urine amount, systolic blood pressure (SBP), diastolic blood pressure (DBP), Kt/V, PET D/Pcr, blood urea nitrogen (BUN), creatinine, phosphorus, calcium, cholesterol, triglyceride, LDL-C, HDL-C, apolipoprotein A, serum ferritin, uric acid, hs-CRP, albumin, prealbumin, hemoglobin, Fe (iron) and TSAT. Total Kt/V was calculated as the sum of renal and peritoneal Kt/V using the urea clearances from 24-h urine and dialysate effluent collections. TSAT was calculated as the ratio of serum iron to transferrin binding capacity. The peritoneal equilibration test (PET), which characterizes peritoneal membrane transport, was performed to calculate the ratio of creatinine concentration in the dialysate to that in plasma after a 4-h dwell (D/Pcr).

### Statistical analysis

Descriptive results of continuous variables are expressed as the mean ± standard deviation (SD), and categorical variables are reported as percentages and numbers. Comparisons between groups were analyzed using one-way analysis of variance and the chi-squared test. The Spearman rank test was performed to estimate the correlations between serum ferritin and other variables. All-cause and cardiovascular mortality rates were assessed by Kaplan–Meier survival analyses with the log-rank test. Univariate Cox proportional hazards regression was applied to estimate the relative risk of possible factors associated with all-cause and cardiovascular mortality. To adjust for confounding factors, multivariate Cox regression analysis with forward regression was performed (factors with *P* < 0.10 in the univariate analysis were added in). A *P* value < 0.05 was considered to indicate statistical significance. All statistical analyses were performed using Statistical Package for the Social Sciences (SPSS) version 20.0 for Windows (SPSS Inc., Chicago, IL, USA).

## Results

### Baseline characteristics of the study subjects

A total of 221 PD patients were included, and the baseline demographic, clinical, and laboratory characteristics are shown in Table 1. The mean age was 53.0 ± 14.1 years, the female/male composition was122/99, and the median follow-up time was 35 (3 ~ 109) months. During the follow-up period, 50 (22.6%) patients died. The underlying kidney diseases were chronic glomerulonephritis (42.1%), diabetic nephropathy (23.1%), hypertensive nephropathy (14.0%), obstructive nephropathy (8.1%), lupus nephritis (7.7%) and others (5.0%).

**Table 1:** Baseline characteristics of all, high ferritin and low ferritin patients

	All patients (*n* = 221)	Ferritin ≥100 μg/L (*n* = 152)		Ferritin < 100 μg/L (*n* = 69)		*P*
		hsCRP > 3 mg/L (*n* = 96)	hsCRP≤ 3 mg/L (*n* = 56)	hsCRP> 3 mg/L (*n* = 45)	hsCRP≤ 3 mg/L (*n* = 24)	
Ferritin, μg/L	291.4 ± 334.1	435.3 ± 384.2	355.4 ± 266.1	42.1 ± 26.0	36.1 ± 24.1	0.000
**Demographics**						
Age, years	53.0 ± 14.1	55.0 ± 14.6	52.5 ± 13.0	52.5 ± 16.1	47.8 ± 10.8	0.308
Male, n (%)	122(55.5)	63 (65.6)	34 (60.7)	22 (48.9)	3 (12.5)	0.000
Body mass index, kg/m2	22.6 ± 4.6	21.9 ± 4.2	22.0 ± 4.7	22.5 ± 3.7	21.3 ± 3.9	0.377
**Causes of ESKD, % (n)**						
Chronic glomerulonephritis	93 (42.1)	30 (31.3)	32 (57.1)	20 (44.4)	11 (45.9)	0.018
Diabetes mellitus	51 (23.1)	21 (21.9)	11 (19.6)	13 (28.9)	6 (25.0)	0.716
Hypertension	31 (14.0)	20 (20.8)	3 (5.4)	7 (15.6)	1 (4.2)	0.027
Obstructive nephropathy	18 (8.1)	9 (9.4)	3 (5.4)	4 (8.9)	2 (8.3)	0.847
Lupus nephritis	17 (7.7)	9 (9.4)	3 (5.4)	1 (2.2)	4 (16.7)	0.143
Others	11 (5.0)	7 (7.2)	4 (7.1)	0 (0.0)	0 (0)	0.154
**Deaths**						
All-cause death, % (*n*)	50 (22.6)	31 (32.3)	8 (14.3)	8 (17.8)	3 (12.5)	0.025
Cardiovascular death, % (n)	23 (10.4)	15 (15.6)	3 (5.3)	3 (6.7)	2 (8.3)	0.163
**Biochemical parameters**						
24 h Urine Amount, mL	1366.8 ± 639.5	1326.5 ± 636.0	1371.9 ± 638.1	1347.0 ± 667.1	1427.8 ± 711.1	0.952
SBP, mmHg	156.0 ± 21.7	157.8 ± 20.5	153.0 ± 24.0	153.0 ± 23.9	159.0 ± 24.0	0.416
DBP, mmHg	87.8 ± 13.4	88.2 ± 12.5	86.5 ± 13.0	87.8 ± 14.8	89.1 ± 15.9	0.871
Kt/V	2.0 ± 0.6	2.0 ± 0.6	2.3 ± 1.0	2.0 ± 0.4	2.0 ± 0.4	0.739
PET D/Pcr	0.7 ± 0.1	0.6 ± 0.1	0.7 ± 0.1	0.7 ± 0.1	0.7 ± 0.1	0.484
BUN, mmol/L	29.6 ± 12.6	29.7 ± 13.8	31.7 ± 12.0	27.2 ± 12.0	29.3 ± 9.4	0.377
Creatinine, μmol/L	790.6 ± 258.0	785.8 ± 282.0	807.2 ± 211.3	812.0 ± 253.2	742.9 ± 273.3	0.731
eGFR, mL/min/1.73 m2	6.0 ± 2.6	6.4 ± 2.7	5.3 ± 2.2	5.6 ± 2.1	6.1 ± 3.4	0.271
Phosphorus, mmol/L	2.0 ± 0.6	2.1 ± 0.7	2.1 ± 0.6	2.0 ± 0.6	2.1 ± 0.5	0.854
Calcium, mmol/L	1.9 ± 0.3	1.9 ± 0.3	2.0 ± 0.3	1.9 ± 0.3	2.0 ± 0.2	0.431
Cholesterol, mmol/L	4.7 ± 1.5	4.7 ± 1.7	4.7 ± 1.3	4.6 ± 1.2	5.4 ± 1.6	0.153
Triglyceride, mmol/L	1.7 ± 1.0	1.6 ± 1.0	1.7 ± 0.9	1.8 ± 1.1	1.7 ± 1.1	0.787
LDL-C, mmol/L	2.9 ± 1.1	2.9 ± 1.2	2.9 ± 1.0	3.1 ± 1.1	3.1 ± 1.1	0.745
HDL-C, mmol/L	1.1 ± 0.3	1.1 ± 0.3	1.1 ± 0.3	1.1 ± 0.3	1.3 ± 0.4	0.067
apolipoproteinA, g/L	1.0 ± 0.2	1.0 ± 0.3	1.1 ± 0.2	1.0 ± 0.2	1.1 ± 0.3	0.319
Uric acid, μmol/L	538.0 ± 140.2	552.0 ± 138.9	550.0 ± 126.6	493.2 ± 136.2	539.5 ± 169.8	0.132
hs-CRP, mg/L	21.0 ± 36.3	37.1 ± 43.2	0.7 ± 0.1	20.3 ± 35.0	1.1 ± 0.8	0.000
Albumin, g/L	32.7 ± 5.4	31.9 ± 5.2	34.3 ± 6.0	32.3 ± 4.6	33.0 ± 5.7	0.067
Prealbumin, g/L	0.3 ± 0.2	0.3 ± 0.1	0.4 ± 0.1	0.4 ± 0.3	0.3 ± 0.1	0.145
Hemoglobin, g/L	82.0 ± 20.0	78.9 ± 21.6	81.0 ± 17.6	84.4 ± 20.8	84.1 ± 15.8	0.427
iPTH, pg/mL	349.0 ± 292.2	307.2 ± 281.3	368.2 ± 238.2	382.9 ± 368.8	391.7 ± 295.6	0.587
TAST, %	0.3 ± 0.2	0.3 ± 0.2	0.4 ± 0.2	0.3 ± 0.2	0.4 ± 0.2	0.407
Fe (iron), μmol/L	48.1 ± 44.1	48.4 ± 49.8	50.7 ± 42.0	44.4 ± 33.0	42.5 ± 40.4	0.909

Values expressed as mean ± SD, or number (percent)

### Comparison of the low and high ferritin groups at baseline

The cutoff value of 100 μg/L was determined based on clinical practice recommendations (Kidney Disease Outcomes Quality Initiative [KDOQI] 2006 [13], Kidney Disease: Improving Global Outcomes [KDIGO] 2012 [14], and The Japanese Society for Dialysis Therapy (JSDT) guidelines [15, 16] and previous research [17]. A standard hsCRP level of 3 mg/L was determined according to the recommendation of the American Heart Association [18]. The patients were divided into the following four groups on the basis of serum ferritin and hsCRP levels: ferritin ≥100 μg/L and hsCRP > 3 mg/L, ferritin ≥100 μg/L and hsCRP ≤3 mg/L, ferritin < 100 μg/L and hsCRP > 3 mg/L, and ferritin < 100 μg/L and hsCRP ≤3 mg/L. As shown in Table 1, patients in the ferritin ≥100 μg/L and hsCRP > 3 mg/L group had a higher proportion of males and higher all-cause mortality (32.3%) and cardiovascular mortality (15.6%). These four groups showed no significant differences in age, BMI, 24-h urine amount, blood pressure, Kt/V, PET, BUN, serum creatinine, phosphorus, calcium, cholesterol, triglyceride, LDL-C, HDL-C, apolipoprotein A, uric acid, albumin, prealbumin, hemoglobin iron and TSAT.

### Correlation between serum ferritin and clinical variables at baseline

The correlation between serum ferritin and clinical parameters was analyzed by Spearman’s correlation method (Table 2). Serum ferritin was significantly correlated with male sex and positively related to BUN and uric acid. However, in the multiple linear regression analysis, including significant variables from the univariate analysis, no clinical parameters were notably correlated with ferritin levels including hsCRP (*P*>0.05).

**Table 2:** Characteristics and correlation between serum ferritin levels and clinical variables at baseline

	Spearman r	*P* value
Age, years	0.015	0.859
Male, *n* (%)	−0.282	0.000
Duration of PD, months	−0.186	0.070
24 h Urine Amount, mL	0.027	0.760
SBP, mmHg	0.043	0.548
DBP, mmHg	0.022	0.762
Kt/V	−0.053	0.819
BUN, mmol/L	0.156	0.020
Creatinine, μmol/L	0.011	0.871
Phosphorus, mmol/L	0.098	0.146
Calcium, mmol/L	0.038	0.580
Cholesterol, mmol/L	−0.065	0.339
Triglyceride, mmol/L	−0.153	0.065
LDL-C, mmol/L	−0.044	0.514
HDL-C, mmol/L	−0.090	0.188
apolipoprotein A, g/L	−0.114	0.171
Uric acid, μmol/L	0.153	0.026
hsCRP, mg/L	0.116	0.088
Albumin, g/L	0.029	0.669
Prealbumin, g/L	0.063	0.388
Hemoglobin, g/L	−0.118	0.083
TAST, %	0.086	0.223
Fe (iron), μmol/L	0.045	0.588

Values expressed as Spearman’s correlation coefficient r

### Survival analysis

**Fig. 1:**
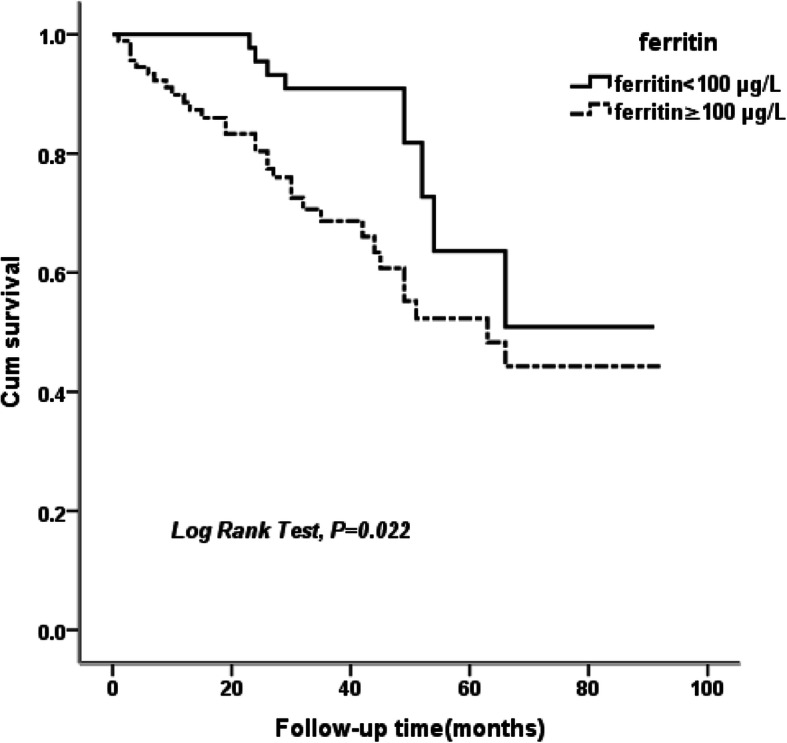
Kaplan-Meier survival curves depicting the survival probability based on serum ferritin under elevated hsCRP levels (> 3 mg/L) among PD patients during follow-up time

**Fig. 2:**
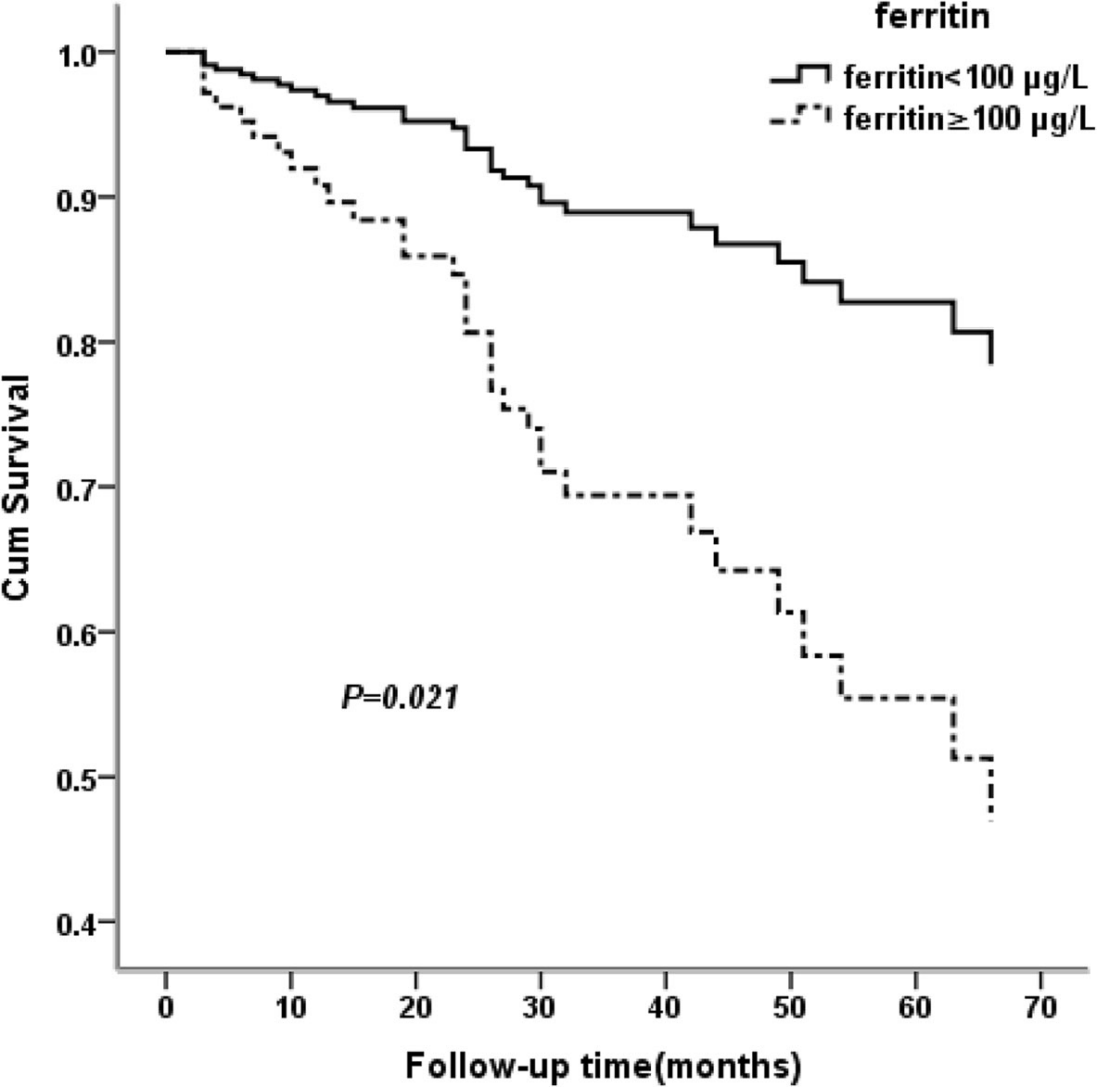
Survival probability with respect to serum ferritin under elevated hsCRP levels (> 3 mg/L) by multivariate cox proportional hazards regression analysis with age, gender and other confounding risk factors adjusted

The ability of serum ferritin to predict mortality during a median follow-up of 35 month was examined. Of the 221 patients with PD, 50 died during follow-up, and the most common cause of death was cardiovascular disease (46.0%), followed by infection (10.0%). Kaplan-Meier curves showed higher mortality among PD patients in the high ferritin group with hsCRP above 3 mg/L (Log-rank test, *P* = 0.022) (Fig. 1)**.** In contrast, no significant difference in mortality was observed among patients with hsCRP levels less than 3 mg/L **(**data not shown**)**. A Cox proportional hazard model was further applied to assess the independent predictors of all-cause and cardiovascular mortality in PD patients. A multivariate analysis was performed after adjusting for age, gender and other confounding factors (factors with *P* < 0.10 in the univariate analysis) under the condition of high hsCRP levels (> 3 mg/L), and serum ferritin level was identified as an independent risk factor for all-cause and cardiovascular mortality (HR: 3.114, *P* = 0.021; HR: 9.382, *P* = 0.032) (Table 3**,** Fig. 2). The statistical power results were 0.9107 and 0.9958 for the multivariate Cox regression analyses of serum ferritin with all-cause and cardiovascular mortality, respectively. However, the correlations of serum ferritin with poor outcome were not statistically significant under the condition of low hsCRP levels (**Supplementary Table****1**). In addition, the multivariate analysis showed that uric acid was associated with all-cause mortality (HR: 1.003, *P* = 0.040), and age was associated with cardiovascular mortality (HR: 2.991, *P* = 0.042) (Table 3).

**Table 3:** Cox proportional hazard analyses of all-cause and cardiovascular mortality under high hsCRP level >3 mg/L

Univariate analysis of mortality				
	All-cause mortality		Cardiovascular mortality	
	HR (95%CI)	*P* value	HR (95%CI)	*P* value
Age, years	1.281 (0.651–2.522)	0.474	1.785 (0.614–5.192)	0.287
Male, n (%)	0.821 (0.431–1.566)	0.550	1.055 (0.416–2.675)	0.910
24 h Urine Amount, mL	1.000 (0.999–1.001)	0.998	1.002 (0.999–1.004)	0.131
SBP, mmHg	1.006 (0.990–1.002)	0.471	1.000 (0.975–1.026)	0.993
DBP, mmHg	0.990 (0.964–1.017)	0.481	0.980 (0.941–1.021)	0.329
Kt/V	1.604 (0.373–2.903)	0.526	0.841 (0.131–5.393)	0.855
PET D/Pcr	0.908 (0.782–63.3)	0.482	0.965 (0.689–1.688)	0.848
BUN, mmol/L	1.006 (0.999–1.012)	0.101	1.009 (1.002–1.015)	0.008
Creatinine, μmol/L	1.000 (0.998–1.001)	0.559	1.000 (0.998–1.001)	0.780
Phosphorus, mmol/L	0.896 (0.558–1.437)	0.648	1.076 (0.582–1.989)	0.815
Calcium, mmol/L	1.170 (0.469–2.917)	0.736	1.015 (0.306–3.985)	0.879
Cholesterol, mmol/L	0.909 (0.743–1.112)	0.353	1.042 (0.805–1.348)	0.757
Triglyceride, mmol/L	1.009 (0.706–1.440)	0.963	1.290 (0.772–2.157)	0.332
LDL-C, mmol/L	0.709 (0.248–2.028)	0.521	0.510 (0.099–2.631)	0.421
HDL-C, mmol/L	0.879 (0.666–1.161)	0.363	0.975 (0.672–1.414)	0.894
apolipoproteinA, g/L	1.095 (0.165–7.246)	0.925	5.919 (0.265–132.113)	0.262
Ferritin, μg/L	2.412 (1.104–5.271)	0.027	2.920 (0.839–10.162)	0.092
Uric acid, μmol/L	1.003 (1.000–1.006)	0.021	1.004 (1.000–1.007)	0.062
hs-CRP, mg/L	1.002 (0.995–1.009)	0.558	1.002 (0.991–1.021)	0.749
Albumin, g/L	0.991 (0.942–1.042)	0.719	1.026 (0.934–1.128)	0.592
Prealbumin, g/L	0.884 (0.583–1.339)	0.560	0.919 (0.609–1.387)	0.686
Hemoglobin, g/L	0.995 (0.982–1.009)	0.515	0.902 (0.982–1.020)	0.902
TAST, %	1.001 (0.986–1.017)	0.849	0.998 (0.974–1.021)	0.846
Fe (iron), μmol/L	0.997 (0.990–1.004)	0.428	1.001 (0.993–1.009)	0.748
Multivariate analysis of mortality				
Ferritin, μg/L	3.114 (1.118–8.162)	0.021	9.382 (1.123–72.581)	0.032
Age, years	–	–	2.991 (1.040–8.598)	0.042
Uric acid, μmol/L	1.003 (1.000–1.006)	0.040	–	–

Values express as hazard ratio (HR) and 95% confidence interval(95%CI)

## Discussion

The present study demonstrates that systemic inflammation has an important effect on the relationship between serum ferritin and the survival of patients undergoing PD. Our results show that a relatively high ferritin level is an independent risk factor for all-cause and cardiovascular mortality among PD patients only in the presence of elevated hsCRP levels.

Serum ferritin has been widely recognized as an acute phase reactant and marker of acute and chronic inflammation independent of its classic role as an iron delivery protein [3, 19]. It is nonspecifically enhanced in several disorders, including liver disease, coronary artery disease, rheumatoid arthritis, and cancer [1, 4, 20, 21]. Additionally, elevated serum ferritin levels have been observed in patients with renal impairment, and these elevated levels are closely related to a higher risk of CKD, a rapid decline in renal function and progression to renal replacement therapy (RRT) [22–24]. Accumulating evidence has demonstrated that hyperferritinemia is significantly associated with poor prognosis, including higher mortality in MHD and PD patients [6, 25–27], although the threshold values at which serum ferritin contributes to negative outcomes differ between MHD and PD patients; these ferritin levels are higher than the demand for erythropoiesis.

Although the mechanisms underlying the association of higher ferritin levels with mortality are incompletely understood, some explanations have been proposed. Serum ferritin leaks from damaged cells, losing most of its iron on the way, and this released iron is in an unliganded form that can negatively impact health and stimulate further cell damage [28]. Moreover, human circulating ferritin can induce oxidative stress by releasing iron with aminolevulinate, a uremic toxin [29]. These results are in accord with our observation that increased ferritin levels are associated with higher mortality in PD patients.

Inflammation plays a pivotal pathogenic role in ESKD, which is caused by multiple factors, including uremic toxins, dialysis, and comorbidities [30, 31]. Researchers recently proposed that inflammation may modify the association between some clinical indicators and the long-term prognosis of ESKD patients [11, 12, 32]. Thus, in this study, we questioned whether the correlation of higher serum ferritin with mortality is linked to systemic inflammation and investigated the optimal serum ferritin level for predicting death when considering inflammation in PD patients.

Currently, there are limited data from which to determine the safe cutoff values for serum ferritin levels to appropriately treat anemia in PD patients without evoking obvious negative. Hur S M et al. showed that elevated serum ferritin levels (> 250 μg/L) was significantly associated with a more rapid RRF decline in patients undergoing PD [27]. CKD patients with ferritin levels above 288 μg/L were more likely to have adverse renal outcomes [24]. In our cohort, a cutoff value for ferritin of 100 μg/L was selected based on the largely opinion-based clinical practice recommendations KDOQI 2006 [13] and KDIGO 2012 [14], which state that supplemental iron should be administered to maintain ferritin levels > 100 μg/L in CKD5 PD patients. Moreover, Kuragano et al. performed a prospective, observational, multicenter study of 1086 Japanese HD patients and found that hyperferritinemia, defined as serum ferritin > 100 μg/L, is a risk factor for cardiovascular disease, infection, hospitalization and death [17].

In this study, mortality was higher among PD patients with serum ferritin values≥100 μg/L than among those with ferritin levels < 100 μg/L **(**Table 1). To ascertain whether systemic inflammation affects the association between serum ferritin and mortality risk, we used Kaplan–Meier survival analyses and Cox survival models to confirm that hyperferritinemia is an independent predictor of all-cause and cardiovascular mortality after adjusting for age, gender and other relevant confounding factors in the hsCRP > 3 mg/L group, but not in the hsCRP < 3 mg/L group. Our study demonstrated a dramatic catalytic effect of inflammation on the association between serum ferritin and unfavorable prognosis in PD patients.

A Ferritin-mediated feed-forward inflammatory loop might explain why serum ferritin has predictive power in PD patients with inflammation. Ferritin molecules are composed of heavy (H) and light (L) chain subunits. Inflammatory cytokines upregulate ferritin synthesis by increasing both the H and L subunits [33], especially the translation of H chain mRNA through direct interactions with the 5′ UTR [34, 35]. A positive feedback loop ensues in which ferritin production downstream of TLR9 activation leads to increased amplification of inflammatory signals [36]. In addition, published data have indicated that persistent inflammation in the uremic milieu might exacerbate the effect of other concurrent risk factors by increasing signaling through the inflammatory cascade and exacerbating both the wasting and vascular calcification processes [12].

Although higher serum ferritin is always related to inflammation under various inflammatory conditions, we did not find a positive correlation between serum ferritin and hsCRP in this study. Some factors may be responsible for this inconsistency. Serum ferritin levels are influenced by multiple factors, especially in ESKD patients, who are always treated with iron supplementation, which significantly increases serum ferritin levels without apparent inflammation. Additionally, in this study, most PD patients were not complicated by acute infection and were sufficiently healthy to undergo percutaneous PD catheter insertion. These abovementioned reasons may explain the lack of a correlation between serum ferritin and hsCRP in this study.

Some limitations need to be considered. First, this was a retrospective, observational study conducted at a single center with a relatively small sample size. Second, we used a single serum ferritin concentration determined at baseline in accordance with similar published studies about the effect of serum ferritin in dialysis patients [27, 37]. However, the mean serum ferritin level has been reported to not change over a 1-year period, indicating consistent levels after the start of PD [27]. Finally, biomarkers of inflammation other than hsCRP, including IL-1, TNF-α and IFN-γ, were not analyzed due to incomplete clinical records; nonetheless, hsCRP is a more sensitive inflammatory biomarker than the others [10].

## Conclusion

In conclusion, a high serum ferritin level (≥ 100 μg/L) was positively associated with a higher risk of all-cause and cardiovascular mortality in patients undergoing PD only in the presence of an elevated hsCRP level (> 3 mg/L). The correlation of serum ferritin with the reduced long-term survival of PD patients should take into consideration systemic inflammation.

## Supplementary information

**Additional file 1: Supplementary Table 1.** Cox proportional hazard analyses of all-cause and cardiovascular mortality with low hsCRP levels (≤3 mg/L)

## Data Availability

The datasets used and/or analyzed during the current study are available from the corresponding author on reasonable request.

## Abbreviations

PD: Peritoneal dialysis
hsCRP: High-sensitivity C-reactive protein
CKD: Chronic kidney disease
ROS: Reactive oxygen species
CRP: C-reactive protein
ESKD: End stage kidney disease
CAPD: Continuous ambulatory peritoneal dialysis
ESA: Erythropoiesis-stimulating agent
BMI: Body mass index
SBP: Systolic blood pressure
DBP: Diastolic blood pressure
PET: Peritoneal membrane transport
BUN: Blood urea nitrogen
TSAT: Transferrin saturation
SD: Standard deviation
KDOQI: Kidney Disease Outcomes Quality Initiative
KDIGO: Kidney Disease: Improving Global Outcomes
HR: Hazard ratio
RRT: Renal replacement therapy
MHD: Maintenance hemodialysis
