# Supplementary material for: Systemic inflammation modulates the ability of serum ferritin to predict all-cause and cardiovascular mortality in peritoneal dialysis patients
Source: BMC Nephrol. 2020 Jun 23;21:237. doi: 10.1186/s12882-020-01892-9 (PMC7310354; doi:10.1186/s12882-020-01892-9)
Supplement: Supplementary file 1 — Additional file 1: Supplementary Table 1. Cox proportional hazard analyses of all-cause and cardiovascular mortality with low hsCRP levels (≤3 mg/L) [file 12882_2020_1892_MOESM1_ESM.pdf]

Supplementary table1: Cox proportional hazard analyses of all-cause and cardiovascular mortality with low hsCRP levels (≤3mg/L)

Univariate analysis of mortality				
	All-cause mortality		Cardiovascular mortality	
	HR (95%CI)	P value	HR (95%CI)	P value
Age, years	0.671(0.158-2.846)	0.588	1.554(0.257-9.375)	0.631
Male, n (%)	1.260(0.383-4.141)	0.704	0.740(0.123-4.437)	0.742
24h Urine Amount, mL	0.999(0.997-1.001)	0.206	0.999(0.996-1.001)	0.345
SBP, mmHg	1.009(0.998-1.031)	0.411	1.017(0.975-1.060)	0.436
DBP, mmHg	1.000(0.954-1.049)	0.990	0.975(0.876-1.085)	0.641
Kt/V	0.151(0.006-3.958)	0.257	0.334(0.022-5.117)	0.431
PET D/Pcr	0.852 (0.303-3.411)	0.852	0.797(0.273-4.722)	0.799
BUN, mmol/L	0.987(0.927-1.052)	0.695	1.023(0.945-1.107)	0.575
Creatinine, µmol/L	1.000(0.998-1.002)	0.999	1.001(0.999-1.003)	0.399
Phosphorus, mmol/L	2.006(0.664-6.058)	0.217	3.089(0.670-14.243)	0.148
Calcium, mmol/L	1.018(0.996-1.040)	0.109	0.182(0.016-2.024)	0.166
Cholesterol, mmol/L	1.112(0.733-1.685)	0.618	1.288(0.736-2.252)	0.375
Triglyceride, mmol/L	2.047(0.994-4.219)	0.052	1.965(0.796-4.852)	0.143
LDL-C, mmol/L	1.565(0.882-2.778)	0.126	1.741(0.791-3.832)	0.169
HDL-C, mmol/L	0.845(0.263-2.720)	0.778	0.759(0.123-4.676)	0.766
apolipoproteinA, g/L	0.147(0.004-5.114)	0.290	0.317(0.004-24.912)	0.606
Ferritin, µg/L	0.886(.234-3.364)	0.859	0.695(0.115-4.197)	0.692
Uric acid, µmol/L	1.003(0.999-1.008)	0.126	1.002(0.995-1.009)	0.613
hs-CRP, mg/L	1.002(0.995-1.009)	0.558	1.441(0.655-3.168)	0.363
Albumin, g/L	1.035(0.945-1.135)	0.459	0.960(0.822-1.121)	0.605
Prealbumin, g/L	0.387(0.018-8.486)	0.547	0.059(0.000-127.102)	0.470
Hemoglobin, g/L	1.016(0.979-1.054)	0.406	0.967(0.915-1.002)	0.232
TAST, %	0.956(0.906-1.009)	0.099	0.944(0.870-1.024)	0.163
Fe (iron), µmol/L	0.975(0.952-0.999)	0.040	0.967(0.930-1.005)	0.090

Values express as hazard ratio (HR) and 95% confidence interval(95%CI).
